# Serum Autoantibodies against LRDD, STC1, and FOXA1 as Biomarkers in the Detection of Ovarian Cancer

**DOI:** 10.1155/2022/6657820

**Published:** 2022-03-01

**Authors:** Yaru Duan, Chi Cui, Cuipeng Qiu, Guiying Sun, Xiao Wang, Peng Wang, Hua Ye, Liping Dai, Jianxiang Shi

**Affiliations:** ^1^School of Basic Medical Sciences, Academy of Medical Sciences, Zhengzhou University, Zhengzhou, 450001 Henan, China; ^2^BGI College & Henan Institute of Medical and Pharmaceutical Sciences, Academy of Medical Sciences, Zhengzhou University, Zhengzhou, 450052 Henan, China; ^3^Henan Key Laboratory of Tumor Epidemiology, College of Public Health, Zhengzhou University, Zhengzhou, 450052 Henan, China; ^4^State Key Laboratory of Esophageal Cancer Prevention & Treatment, Zhengzhou University, Zhengzhou, 450052 Henan, China

## Abstract

**Purpose:**

This study is aimed at evaluating serum autoantibodies against four tumor-associated antigens, including LRDD, STC1, FOXA1, and EDNRB, as biomarkers in the immunodiagnosis of ovarian cancer (OC).

**Methods:**

The autoantibodies against LRDD, STC1, FOXA1, and EDNRB were measured using an enzyme-linked immunosorbent assay (ELISA) in 94 OC patients and 94 normal healthy controls (NHC) in the research group. In addition, the diagnostic values of different autoantibodies were validated in another independent validation group, which comprised 136 OC patients, 136 NHC, and 181 patients with benign ovarian diseases (BOD).

**Results:**

In the research group, autoantibodies against LRDD, STC1, and FOXA1 had higher serum titer in OC patients than NHC (*P* < 0.001). The area under receiver operating characteristic curves (AUCs) of these three autoantibodies were 0.910, 0.879, and 0.817, respectively. In the validation group, they showed AUCs of 0.759, 0.762, and 0.817 and sensitivities of 49.3%, 42.7%, and 48.5%, respectively, at specificity over 90% for discriminating OC patients from NHC. For discriminating OC patients from BOD, they showed AUCs of 0.718, 0.729, and 0.814 and sensitivities of 47.1%, 39.0%, and 51.5%, respectively, at specificity over 90%. The parallel analyses demonstrated that the combination of anti-LRDD and anti-FOXA1 autoantibodies achieved the optimal diagnostic performance with the sensitivity of 58.1% at 87.5% specificity and accuracy of 72.8%. The positive rate of the optimal autoantibody panel improved from 62.4% to 87.1% when combined with CA125 in detecting OC patients.

**Conclusion:**

Serum autoantibodies against LRDD, STC1, and FOXA1 have potential diagnostic values in detecting OC.

## 1. Introduction

Ovarian cancer (OC) remains the deadliest cancer in women worldwide. There were an estimated 22,530 new cases and 13,980 deaths from OC in the United States in 2019, making it the leading cause of cancer deaths among gynecologic malignancies [1]. Although advances in OC treatment significantly improved the five-year survival of OC patients over the last three decades, the overall cure rate remained less than 30% [2]. When the lesion is restricted within the ovaries, up to 90% of patients can be cured following routine surgery and chemotherapy, and the five-year survival rate is approximately 50% for disease limit to the pelvis (stage II) with treatment strategies [3]. Meanwhile, the five-year survival rate for the disease beyond the pelvis (stage III-IV) is less than 20% [4]. However, due to the absence of specific symptoms at the early stage, the vast majority of OC patients were diagnosed at an advanced stage (stage III or stage IV), and only 20% of OC patients were initially diagnosed at an early stage [5]. Hence, the quest for applicable and reliable biomarkers for early detection is critical in improving the clinical outcomes of OC patients.

Carbohydrate antigen 125 (CA125) and transvaginal ultrasonography (TVUs) are typically used in clinical settings and can detect some OC patients at early stage. Human epididymal protein (HE4) can detect a small fraction of OC patients missed by CA125 [3]. The study showed that the diagnostic value of CA125 and HE4 with areas under the receiver operating characteristic curves (AUCs) were 0.78 and 0.76, respectively, for the discrimination between benign and stage I OC [6]. However, these serum markers are not ideal biomarkers in the early detection of OC due to their limited sensitivity [7]. TVUs lack adequate sensitivity and yield a high false-positive rate [8, 9]. Therefore, the discovery of biomarkers for the early detection of OC is of considerable importance. Tumor-associated antigens (TAAs) are a kind of protein aberrantly expressed in cancer, which can elicit an autoimmune response and the production of corresponding autoantibodies accordingly [10]. Autoantibodies against TAAs are more stable and have a relatively higher titer in serum or plasma due to the amplification effect of the immune system compared with their corresponding TAAs [11].

LRDD, also known as PIDD, which contains two protein interaction domains, is a leucine-rich repeat and death domain-containing protein [12]. It is induced by TP53 and acts as a molecular switch to promote cell survival or apoptosis [13]. A previous study showed that LRDD was the downstream target gene of TP53, and it could increase the TP53 protein expression in a positive feedback loop [14, 15]. Stanniocalcin-1 (STC1) is a glycoprotein hormone, and it is involved in regulating calcium and phosphate homeostasis, which was initially discovered in bony fishes [16]. Mammalian STC1 is expressed in various tissues, with the highest levels in the ovary, prostate, kidney, lung, colon, and thyroid [17, 18]. Studies show that STC1 functions as a proto-oncogene and participates in the biological process of tumorigenesis [19]. Forkhead-box A1 (FOXA1), also known as hepatocyte nuclear factor 3-a (HNF3a), is a central regulator in the normal development of several endoderm-derived tissues [20]. FOXA1 can directly bind to the DNA and open the chromatin to enhance the transcription; so, it is described as a pioneer transcription activator [21]. It has been confirmed that FOXA1 is positively expressed in OC tissue, and it is involved in the pathogenesis and development of OC [22]. Endothelin receptor type B (EDNRB) is a member of the family of G-protein-coupled receptors, which plays a vital role in tumor cell proliferation, migration, and lymph angiogenesis via combination with endothelin-1 [23, 24]. Aberrant methylation of EDNRB and decreased expression of mRNA were identified in various cancers [25].

There is growing evidence that these four proteins are associated with the occurrence and development of cancer. However, studies on the feasibility that their corresponding autoantibodies serve as biomarkers of cancer are still sparse, especially for OC. Therefore, this study is aimed at evaluating the diagnostic value of their corresponding autoantibodies in OC detection.

## 2. Materials and Methods

### 2.1. Serum Samples

The research group comprised sera from 94 OC patients and 94 normal healthy controls (NHC), respectively. Sera from OC patients were obtained from a tertiary level hospital (Zhengzhou, China) from March 1, 2011, to April 30, 2012, and sera of NHC were collected from the cardiovascular disease investigation project (Henan Province, China) without the benign ovarian disease (BOD) or disease associated with the immune system. The validation group comprised 453 sera from 136 OC patients with histological confirmation, 136 NHC, and 181 patients with BOD. In the validation group, sera from OC and BOD patients were obtained from a tertiary level hospital (Zhengzhou, China) from July 1, 2017, to April 30, 2018, and sera from NHC was derived from the biobank of Henan Key Laboratory of Tumor Epidemiology. The collection of all serum samples followed standardized protocol, and serum samples were stored at −80°C until further use. All patients signed written informed consent, and the current study was approved by the Institutional Review Board of Zhengzhou University.

### 2.2. Enzyme-Linked Immunosorbent Assay (ELISA)

Autoantibodies against LRDD, STC1, EDNRB, and FOXA1 in human serum samples were measured by ELISA, and the detailed protocol were described previously [26]. In brief, three recombinant proteins (Cloud-Clone, china) were diluted to the optimal concentration (0.125 *μ*g/mL, respectively) using a coating buffer. Recombinant proteins (50 *μ*l/well) were added to the 96-well ELISA plates, incubated at 4°C overnight. 96-well plates were blocked with 2% bovine serum albumin (BSA) at 4°C overnight to reduce the nonspecific reaction. Then, PBST (0.01% Tween 20 in phosphate-buffered saline) was used to wash the plates three times. Next, human serum samples diluted at 1 : 100 in 1% BSA were added to the antigen-coated 96-well plates. The plates were incubated at 37°C for 1 hour. Following by five times of wash with PBST, the secondary antibody goat anti-human IgG horseradish peroxidase-conjugated (HRP) diluted at 1 : 5000 was added to each well for 1 hour incubated at 37°C followed by washing five times with PBST. The solution of 3,3′,5,5′-tetramethylbenzidine (TMB)-H_2_O_2_-urea was used as detecting reagents. 25 *μ*l of 2 M sulfuric acid served as the stopping solution. The optical density (OD) values were measured at 450 nm and 620 nm with a multimode plate reader (PerkinElmer envision 2105, USA). For quality control, six fixed human serum samples were used as references to mitigate batch effects between plates, and the last two wells of the last column of each plate served as blank controls, respectively.

### 2.3. Statistical Analysis

The differences of autoantibodies between OC and NHC were assessed using independent sample *t*-test and Mann–Whitney *U* test. One-way analysis of variance (ANOVA) and the Kruskal-Wallis H test were applied to compare the differences in more than two groups. The cutoff values (the OD value corresponding to the maximal Youden index at specificity over 90%) were used to determine a positive reaction for each autoantibody. The Chi-square test was conducted to compare the positive rates between the OC group and NHC group. The receiver operating characteristic (ROC), sensitivity, specificity, negative likelihood ratio (LR−), positive likelihood ratio (LR+), and accuracy rate were performed to assess the diagnostic performance of each autoantibody. Two-tailed *P* values less than 0.05 were considered statistically different. Statistical analysis was carried out by SPSS 26.0 and GraphPad prism software 8.0.

## 3. Results

### 3.1. Characteristics of the Study Population

In the present study, two independent groups were designed to investigate the diagnostic values of autoantibodies against 4 TAAs for OC in 641 serum samples by ELISA. The research group enrolled 94 OC patients with age of 54.2 ± 12.0 years (range from 21 to 83 years) and 94 NHC with age of56.9 ± 12.7 years (range from 27 to 83 years). The validation group included 136 OC patients with age of 51.9 ± 11.9 years (range from 16 to 74 years), 136 NHC with age of 50.2 ± 11.6 years (range from 20 to 83 years), and 181 BOD patients with age of 35.8 ± 10.6 years (range from 20 to 68 years).  Table 1 shows the clinicopathological features of the study population for both research and validation groups.

### 3.2. Serum Titers and Diagnostic Values of Autoantibodies against 4 TAAs in the Research Group

**Figure 1:**
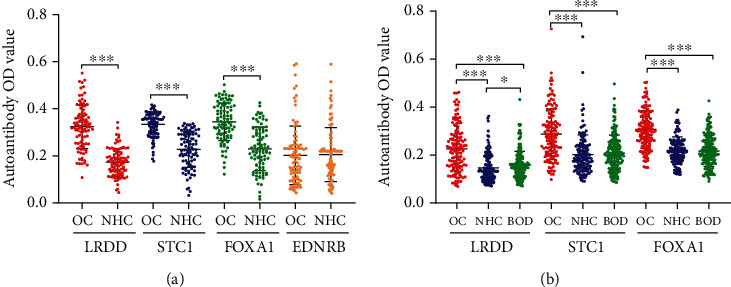
Scatter plots of the optical density (OD) value for autoantibodies in the research and validation group. (a) research group (b) validation group. ^∗∗∗^*P* < 0.001, ^∗^*P* < 0.05. OC: ovarian cancer; NHC: normal healthy control; BOD: benign ovarian diseases. Lines represented median and quartile range.

**Figure 2:**
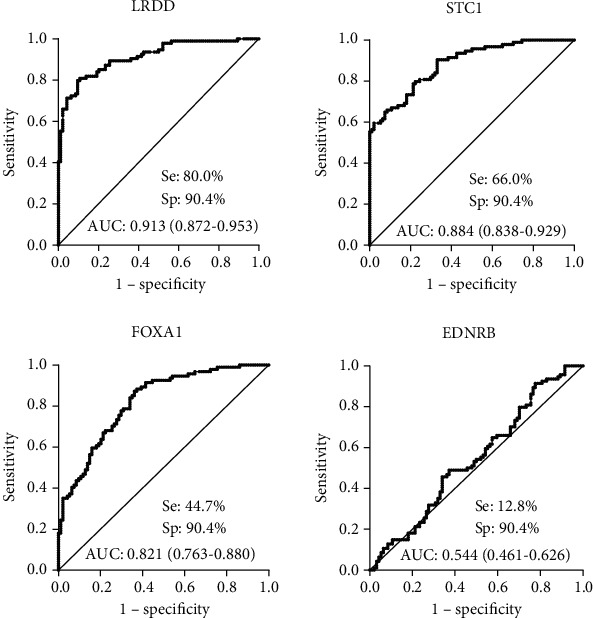
Diagnostic performances of autoantibodies against LRDD, STC1, FOXA1, and EDNRB in the research group. Se: sensitivity; Sp: specificity; AUC: area under the receiver operating characteristic curve.

The serum autoantibodies were measured by ELISA in 94 patients diagnosed with OC and 94 age-matched NHC to assess whether autoantibodies against LRDD, STC1, EDNRB, and FOXA1 can be used as biomarkers in the detection of OC. Autoantibodies against LRDD, STC1, and FOXA1 were significantly higher in OC patients than in NHC (*P* < 0.001). In contrast, the autoantibody against EDNRB in OC patients and NHC did not show a statistical difference (*P* > 0.05) (Figure 1(a)). ROC curves were plotted the to investigate the diagnostic efficacy of autoantibodies against 4 TAAs. As shown in Figure 2, the autoantibodies against LRDD, STC1, and FOXA1 showed good diagnostic performance for distinguishing OC patients from NHC with the area under ROC curves (AUCs) of 0.913, 0.884, and 0.821, respectively. Hence, further validation was applied to these three autoantibodies.

### 3.3. Diagnostic Performance of Anti-LRDD, Anti-STC1, and Anti-FOXA1 Autoantibodies in the Validation Group

**Figure 3:**
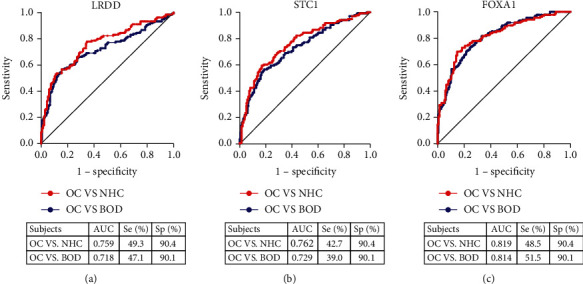
Diagnostic performances of autoantibodies against LRDD, STC1, and FOXA1 in the validation group. The red symbol represents the performance of each autoantibody in distinguishing between OC and NHC, and the blue symbol represents the performance of each autoantibody to distinguish between OC and BOD; OC: ovarian cancer; NHC: normal healthy control; BOD: benign ovarian disease; Se: sensitivity; Sp: specificity; AUC: area under the receiver operating characteristic curve.

**Figure 4:**
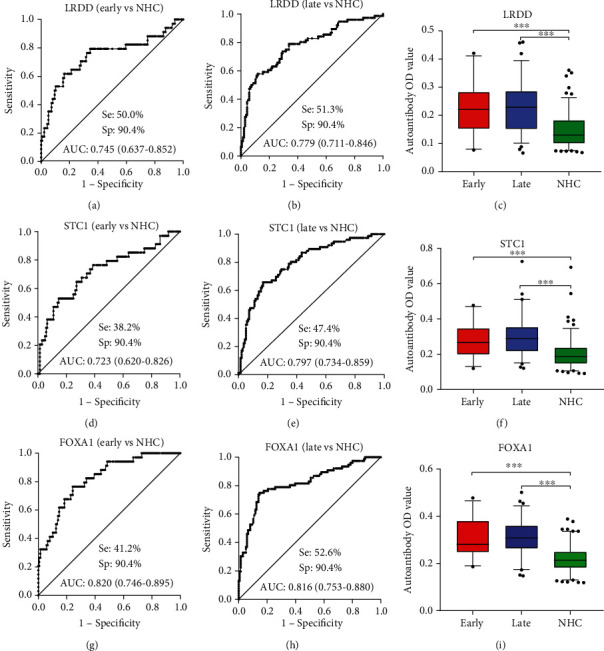
Diagnostic performances of autoantibodies against LRDD, STC1, and FOXA1 for the discrimination of NHC and patients with early-stage or late stage. Box plots present the autoantibodies OD value among early stage, late-stage, and NHC in the validation group. The lines on the boxes represent 95, 75, 50, 25, and 5 percentiles from top to bottom. Whiskers show points greater than 95% quantile and less than 5% quantile; ^∗∗∗^*P* < 0.001. NHC: normal healthy control; Se: sensitivity; Sp: specificity; AUC: area under the receiver operating characteristic curve.

Serum levels of anti-LRDD, anti-STC1, and anti-FOXA1 autoantibodies were measured in another independent group of 136 OC patients, 136 NHC, and 181 BOD patients. The median levels and interquartile ranges of serum autoantibodies in the research and validation group are present in Table 2. Serum autoantibodies against LRDD, STC1, and FOXA1 significantly increased in OC patients compared to NHC and BOD patients (*P* < 0.001). The serum titer of anti-LRDD autoantibody was higher in BOD patients than that in NHC (*P* < 0.05), while no statistical differences were found between BOD patients and NHC for anti-STC1 and anti-FOXA1 autoantibodies (*P* > 0.05) (Figure 1(b)). Subsequently, we performed the ROC curves to evaluate the diagnostic values of these three autoantibodies in the validation group. The AUCs of anti-LRDD, STC1, and FOXA1 autoantibodies were 0.759, 0.762, and 0.819, respectively (Figure 3). When BOD patients were used as controls, serum autoantibodies against LRDD, STC1, and FOXA1 showed AUCs of 0.718, 0.729, and 0.814 in discriminating OC and BOD patients, respectively (Figure 3). A significant difference was found between NHC and early or late-stage OC for anti- LRDD, STC1, and FOXA1 autoantibodies (*P* < 0.001, Figure 4). The potential diagnostic performance of the three autoantibodies was further assessed in different stages of OC. None of the AUCs showed a significant difference between early-stage and late-stage OC (Figure 4).

### 3.4. Positive Rates of Anti-LRDD, STC1, and FOXA1 Autoantibodies in OC and NHC

The OD value corresponding to the maximal Youden index at specificity over 90% was used as the cutoff value to determine a positive reaction for each autoantibody. As shown in Table 3, the sensitivities of anti-LRDD, STC1, and FOXA1 autoantibodies were 79.79%, 65.96%, and 44.68%, respectively, in the research group. In the validation group, the sensitivities of anti-LRDD, STC1, and FOXA1 autoantibodies were 49.26%, 42.65%, and 48.53%, respectively. However, the sensitivity was relatively low for individual autoantibodies at above 90% specificity. We attempted to enhance the sensitivity of the assay using a panel of autoantibodies, and combinational analyses were initiated with anti-LRDD, which showed the highest sensitivity in the three autoantibodies. As shown in Table 4, the sensitivity increased to 59.56% with successive addition of all the three autoantibodies, and the specificity slightly dropped from 90.44% to 87.50%. The maximum YI and accuracy rate from a panel comprising anti-LRDD and anti-FOXA1 autoantibodies reached 0.46 and 72.79%, respectively.

### 3.5. Association between Positive Rates of Autoantibodies and Clinical Characteristics of OC Patients

To compare the positive rates of autoantibody between OC patients with different clinical characteristics, OC patients were classified into different subgroups according to age, family history of tumor, TNM stage, tumor size, lymph node metastasis, and distant metastasis. As shown in Table 5, the positive rate of anti-LRDD autoantibody in OC patients without distant metastasis was significantly higher than patients with distant metastasis (*P* = 0.034). However, no significant differences were found in the positive rates of two individual autoantibodies against STC1, FOXA1, and optimal autoantibody panel in OC patients with different clinicopathological characteristics (all *P* > 0.05). The positive rates appeared to be slightly higher in OC patients at late-stage and with a family history of cancer than OC patients at an early stage and without a family history of tumor, respectively.

### 3.6. Combination of Optimal Autoantibody Panel and CA125 in OC Detection

Eighty-five out of 136 patients in the research group had detailed information on CA125. The positive rates of CA125 and the optimal autoantibody panel alone were 60.0% and 62.4% in OC patients, respectively. However, when we combined the optimal autoantibodies panel with CA125, the positive rate in OC patients increased to 87.1% (Table 6). Further analysis revealed that the combination of optimal autoantibody panel and CA125 was significantly superior to the CA125 or the autoantibody panel alone in detecting OC (*P* < 0.001).

## 4. Discussion

The improvement of survival outcomes in OC is substantially determined by the timely diagnosis and appropriate treatment [27, 28]. To date, the early detection of OC has been hindered by the paucity of effective serum biomarkers. Previous studies have shown that the elevation of anti-TP53 autoantibody levels provided the first biomarker lead time over CA125 to diagnose preclinical diseases in a fraction of cases [29]. This study evaluated the diagnostic value of three biomarkers, including anti-LRDD, anti-STC1, and anti-FOXA1 autoantibodies, in detecting OC. The sensitivity ranged from 42.65% to 49.26% for individual autoantibody at above 90% specificity. The serum levels of these three autoantibodies were significantly higher in OC patients than that in NHC and BOD patients. Besides, the AUC ranged from 0.759 to 0.819, which indicated that autoantibodies against LRDD, STC1, and FOXA1 could be used as biomarkers in detecting OC. An optimal panel of anti-LRDD and anti-FOXA1 autoantibodies was identified. The addition of this autoantibody panel to CA125 achieved a higher positive rate in detecting OC than the use of CA125 or the panel of two autoantibodies alone.

Tumorigenesis is a complex process that involves multigenetic alternations [30]. Oncogenic transformation is due in part to the accumulation of DNA damage [15]. LRDD serves a prominent role in response to DNA damage by mediating the transcription factor NF-kappa-B(NF-*κ*B) activation [31]. Berube and colleagues confirmed that the expression of LRDD could be detected in the nuclear and cytoplasmic fractions of mouse and human cell lines, function as the component of the DNA damage, or genotoxic stress response pathway [14]. Bradley et al. found that the expression of LRDD showed a wide range of oral squamous cell carcinoma, and the expression is extremely high in the tumor with p53 mutation [32]. Accumulating evidence presented that the broad expression of LRDD can be detected in non-small-cell lung cancer tissues [33]. There was a correlation between the high expression of LRDD and clinical data, such as tumor size, tumor stage, and lymph node metastasis. Patients with the high LRDD expression were associated with poor survival [33]. Mounting researches indicated that the higher expression of STC1 was associated with OC, breast cancer, and hepatocellular carcinoma [34–36]. It is noteworthy that the roles of STC1 are intricate in OC and breast cancer. The occurrence of OC and breast cancer is related to the down-regulation of STC1 after losing BRCA1 function [37, 38]. It has been shown that STC1 played a crucial role in promoting tumor metastasis, invasion, tumor cell proliferation, and antiapoptosis via participating in multiple signal pathways associated with cancer, including JNK/c-Jun NF-*κ*B, cyclin E/CDK-2, and ERK1/2 signal pathways [39–42]. Besides, STC1 also could enhance tumor angiogenesis via activation of the VEGF/VEGFR-2 signal pathway [43]. One study suggested that STC1 was positively regulated by desumoylated progesterone receptor in the absence of ligand for the breast cancer cells [37]. It has been demonstrated that STC1 exhibited significant clinical value in the diagnosis, prognosis, and pathological parameters for many kinds of cancer patients [43]. Therefore, STC1 holds promise as a biomarker in the early diagnosis of cancer. FOXA1 is regarded as an oncogene that involves in the tumorigenesis and progression of hormone-dependent cancers [44]. Meanwhile, FOXA1 also has a tumor-suppressive function by suppressing the PI3K signaling pathway, a potential cancer therapeutic target [45]. Mutations in the FOXA1 gene have been recurrently reported in prostate cancer, ER-positive breast cancer, and liver cancer [44, 46, 47]. Increasing studies substantiated that the FOXA1 overexpression can promote tumor metastasis, invasion, and proliferation, particularly in several hormone-independent cancers [47]. In salivary duct carcinoma and bladder cancer, patients with high FOXA1 expression levels are associated with favorable clinical survival outcomes [48, 49]. Data available in immunohistochemical studies suggested that the expression of FOXA1 in OC and salivary duct carcinoma tissue is significantly higher than that in normal tissue, and it may be a potential biomarker for cancer detection [48, 50]. Although these three proteins were proven to have an important effect on the onset of cancer, few studies have investigated the values of these proteins as biomarkers in the diagnosis of cancer.

In the current study, this is the first time to evaluate the diagnostic value of anti-LRDD, anti-STC1, and anti- FOXA1 autoantibodies in OC. There is a significant difference between the OC and the two control groups (NHC and BOD groups). However, the sensitivity for a single autoantibody is limited at over 90% specificity. Therefore, the combinational utilization of autoantibodies could enhance the sensitivity without significantly compromising the specificity. Notably, the parallel combination of anti-LRDD and anti- FOXA1 autoantibodies achieved a sensitivity of 58.09% at the specificity of 87.50%, and the accuracy rate was 72.79%. Wang et al. analyzed 132 OC and 147 NHC, and they showed the sensitivity of 61.4% at the specificity of 85.0% in OC by the parallel combination of nine autoantibodies (autoantibodies against p53, c-MYC, p90, p62, AHSG, and 14-3-3 zeta, RalA, Koc, P16) [51]. Li et al. reported that a panel comprising nine autoantibodies against survivin, p53, p16, cyclin B1, cyclin D1, cyclin A, cyclin E, Koc, and IMP1, P62, CDK2, P90, and c-MYC achieved a sensitivity of 62.5% at 85.4% specificity in the detection of OC [52]. In our study, the panel comprising two autoantibodies was more cost-effective than the panels from the two studies mentioned above. Besides, the results from these two studies have been constrained by lacking an independent validation group compared to our present study.

We also examined the positive rates of autoantibody between different subgroups divided by different clinical characteristics. There was no significant relationship between positive rates of autoantibody and clinical characteristics in OC patients. This may be due to the limited serum samples and incomplete patient data used in this study. Therefore, in the later rounds of the study, there is a need to expand the sample size and collect more detailed clinical characteristics of the cases for further exploring. For most OC patients presenting at a late stage at the time of diagnosis, clinical blood specimens before the diagnosis were unavailable. So, the main limitation of this study is that it was a retrospective study. We focus solely on the identification and validation phase of biomarkers. Further large-scale prospective investigations are required to confirm the diagnostic values of these autoantibodies.

In conclusion, this study indicates that anti-LRDD, anti-STC1, and anti-FOXA1 autoantibodies have high diagnostic values and may complement other serological biomarkers for OC detection. The combination of anti-LRDD and anti-FOXA1 autoantibodies acquired higher sensitivity of detection in OC patients. The combinational utilization of CA125 and anti-LRDD, anti-FOXA1 autoantibodies is promising in detecting OC in the clinical setting. However, the combination of autoantibodies remains to be investigated before future clinical implementation.

## Figures and Tables

**Table 1:** The clinicopathological features of the study population for both research and validation group.

	Research group		Validation group		
	OC (*n* = 94)	NHC (*n* = 94)	OC (*n* = 136)	NHC (*n* = 136)	BOD (*n* = 181)
Age (year)					
Range	21-83	27-83	16-74	20-83	20-68
Mean ± SD	54.2 ± 12.0	56.9 ± 12.7	51.9 ± 11.9	50.2 ± 11.6	35.8 ± 10.6
Gender	Female	Female	Female	Female	Female
FIGO stage					
I-II	3 (3.2)		33 (24.3)		
III-IV	48 (51.1)		77 (56.6)		
Unknown	43 (45.7)		26 (19.1)		
Histologic type					
Epithelial tumor	55 (58.5)		111 (81.6)		
Sexual cord interstitial tumor	3 (3.19)		9 (6.6)		
Germ cell tumor	0(0.00)		5(3.7)		
Unknown	35 (37.2)		11(8.1)		
Family history					
No	90 (95.7)		81 (59.6)		
Yes	0 (0.00)		36 (26.5)		
Unknown	4 (4.3)		19 (14.0)		
Lymph node metastasis					
No	6 (6.4)		48 (35.3)		
Yes	14 (14.9)		49 (36.0)		
Unknown	74 (78.7)		39 (28.7)		
Distant metastasis					
No	40 (42.6)		35 (25.7)		
Yes	14 (14.9)		50 (36.8)		
Unknown	40 (42.6)		51 (37.5)		

Abbreviations: OC: ovarian cancer; NHC: normal healthy controls; BOD:benign ovarian diseases.

**Table 2:** The median levels and interquartile ranges of serum autoantibodies in the research and validation group.

TAAb	Median ± IQR		Median ± IQR		
	OC (*n* = 94)	NC (*n* = 94)	OC (*n* = 136)	BOD (*n* = 136)	NC (*n* = 181)
LRDD	0.324 ± 0.125	0.172 ± 0.081	0.219 ± 0.134	0.275 ± 0.066	0.133 ± 0.081
STC1	0.344 ± 0.078	0.232 ± 0.120	0.275 ± 0.134	0.202 ± 0.092	0.186 ± 0.089
FOXA1	0.343 ± 0.123	0.223 ± 0.151	0.302 ± 0.109	0.211 ± 0.086	0.214 ± 0.067
EDNRB	0.193 ± 0.140	0.195 ± 0.110			

OC: ovarian cancer; NHC: normal healthy control; BOD: benign ovarian diseases. IQR: interquartile.

**Table 3:** Diagnostic value of autoantibody against LRDD, STC1, FOXA1, and EDNRB in human sera by ELISA in the research and validation group.

TAAb	Se (%)	Sp (%)	YI	LR+	LR-	PPV (%)	NPV (%)	Accuracy (%)	*P*
Research group									
LRDD	79.79	90.43	0.70	8.33	0.22	89.29	81.73	85.11	<0.001
STC1	65.96	91.49	0.57	7.75	0.37	88.57	72.88	78.72	<0.001
FOXA1	44.68	90.43	0.35	4.67	0.61	82.35	62.04	67.55	<0.001
EDNRB	12.77	91.49	0.04	1.5	0.95	60.00	51.19	52.13	0.778
Validation group									
LRDD	49.26	90.44	0.40	5.15	0.56	83.75	64.06	69.85	<0.001
STC1	42.65	91.91	0.35	5.27	0.62	84.06	61.58	67.28	<0.001
FOXA1	48.53	91.18	0.40	5.50	0.56	84.62	63.92	69.85	<0.001

Abbreviations: OC: ovarian cancer; NHC: normal healthy controls; Se: sensitivity; Sp: specificity; YI: Youden index; LR+: positive likelihood ratio; LR−: negative likelihood ratio; PPV: positive predictive value; NPV: negative predictive value.

**Table 4:** Diagnostic value of the combinations of autoantibodies.

Panel of TAAbs	Positive, No. (%)		Se, (%)	Sp (%)	YI	LR+	LR-	PPV (%)	NPV, (%)	Accuracy (%)
	OC (*n* = 136)	NHC (*n* = 136)								
LRDD	67 (49.26)	13 (9.56)	49.26	90.44	0.40	5.15	0.56	83.75	64.06	69.85
LRDD, FOXA1	79 (58.09)	17(12.50)	58.09	87.50	0.46	4.65	0.48	82.30	67.61	72.79
LRDD, FOXA1, STC1	81 (59.56)	20 (14.71)	59.56	85.29	0.45	4.05	0.47	80.20	67.84	72.43

Abbreviations: OC: ovarian cancer; NHC: normal healthy controls; Se: sensitivity; Sp: specificity; YI: Youden index; LR+: positive likelihood ratio; LR−: negative likelihood ratio; PPV: positive predictive value; NPV: negative predictive value.

**Table 5:** Subgroup analysis of autoantibody level and clinical characteristics of OC patients.

Variables	*n*	Anti-LRDD		Anti-STC1		Anti-FOXA1		Anti-LRDD or anti-FOXA1	
		Positive (%)	*P*	Positive (%)	*P*	Positive (%)	*P*	Positive (%)	*P*
Age (year)									
<50	52	23 (44.2)		22 (42.3)		27 (51.9)		29 (55.8)	
≥50	84	43 (51.2)	0.430	35 (41.7)	0.941	38 (45.2)	0.448	50 (59.5)	0.666
Family history of tumor									
No	81	39 (48.1)		34 (42.0)		39 (48.1)		48 (59.3)	
Yes	36	20 (55.6)	0.460	18 (50.0)	0.420	20 (55.6)	0.460	23 (63.9)	0.636
TNM stage									
Early stage (I + II)	34	17 (50.0)		13 (38.2)		15 (44.1)		19 (55.9)	
Late stage (III + IV)	76	39 (51.3)	0.898	36 (47.4)	0.373	40 (52.6)	0.409	46 (60.5)	0.647
Tumor size									
<5 cm	9	4 (44.4)		5 (44.4)		5 (55.6)		5 (55.6)	
≥5 cm	33	15 (45.5)	1.000	13 (39.4)	1.000	14 (42.4)	0.746	18 (54.5)	1.000
Lymph node metastasis									
Positive	49	23 (46.9)		21 (42.9)		25 (51.0)		29 (59.2)	
Negative	48	23 (47.9)	0.923	18 (37.5)	0.591	22 (45.8)	0.609	27 (56.3)	0.770
Distant metastasis									
No	35	20 (57.1)		15 (42.9)		19 (54.3)		24 (68.6)	
Yes	50	17 (34.0)	0.034	18 (36.0)	0.523	19 (38.0)	0.137	26 (52.0)	0.127

**Table 6:** Comparison of the positive rate of different combination of biomarkers in OC.

Group	Positive	Negative	^∗^ *P*	#*P*
CA125	51 (60.0)	34 (40.0)		
Optimal panel	53 (62.4)	32 (37.6)		
CA125 + optimal panel	74 (87.1)	11 (12.9)	<0.001	<0.001

^∗^ *P* represents the comparison of positive rate between CA125 and combination of CA125 and optimal panel. ^#^*P* represents the comparison of positive rate between the optimal panel and combination of CA125 and optimal panel.

## Data Availability

The datasets generated and/or analyzed during the current study are available from the corresponding author on reasonable request.

## References

[1] Siegel R. L., Miller K. D., Jemal A. (2019). Cancer statistics, 2019. *CA: a Cancer Journal for Clinicians*.

[2] Yang W. L., Lu Z., Bast R. C. (2017). The role of biomarkers in the management of epithelial ovarian cancer. *Expert Review of Molecular Diagnostics*.

[3] Elias K. M., Guo J., Bast R. C. (2018). Early detection of ovarian cancer. *Hematology/Oncology Clinics of North America*.

[4] Aletti G. D., Dowdy S. C., Podratz K. C., Cliby W. A. (2007). Relationship among surgical complexity, short-term morbidity, and overall survival in primary surgery for advanced ovarian cancer. *American Journal of Obstetrics and Gynecology*.

[5] Ma Y., Wang X., Qiu C. (2021). Using protein microarray to identify and evaluate autoantibodies to tumor-associated antigens in ovarian cancer. *Cancer Science*.

[6] Landolfo C., Achten E. T. L., Ceusters J. (2020). Assessment of protein biomarkers for preoperative differential diagnosis between benign and malignant ovarian tumors. *Gynecologic Oncology*.

[7] Buys S. S., Partridge E., Greene M. H. (2005). Ovarian cancer screening in the prostate, lung, colorectal and ovarian (PLCO) cancer screening trial: findings from the initial screen of a randomized trial. *American Journal of Obstetrics and Gynecology*.

[8] Skates S. J., Greene M. H., Buys S. S. (2017). Early detection of ovarian cancer using the risk of ovarian cancer algorithm with frequent CA125 testing in women at increased familial risk – combined results from two screening trials. *Clinical Cancer Research*.

[9] Menon U., Gentry-Maharaj A., Hallett R. (2009). Sensitivity and specificity of multimodal and ultrasound screening for ovarian cancer, and stage distribution of detected cancers: results of the prevalence screen of the UK collaborative trial of ovarian cancer screening (UKCTOCS). *The Lancet Oncology*.

[10] Tan E. M., Zhang J. (2008). Autoantibodies to tumor-associated antigens: reporters from the immune system. *Immunological Reviews*.

[11] Li P., Shi J. X., Dai L. P. (2016). Serum anti-MDM2 and anti-c-Myc autoantibodies as biomarkers in the early detection of lung cancer. *Oncoimmunology.*.

[12] Telliez J. B., Bean K. M., Lin L. L. (2000). LRDD, a novel leucine rich repeat and death domain containing protein. *Biochimica et Biophysica Acta*.

[13] Lin Y., Ma W., Benchimol S. (2000). Pidd, a new death-domain-containing protein, is induced by p 53 and promotes apoptosis. *Nature Genetics*.

[14] Berube C., Boucher L. M., Ma W. (2005). Apoptosis caused by p53-induced protein with death domain (PIDD) depends on the death adapter protein RAIDD. *Proceedings of the National Academy of Sciences of the United States of America*.

[15] Cuenin S., Tinel A., Janssens S., Tschopp J. (2008). p53-induced protein with a death domain (PIDD) isoforms differentially activate nuclear factor-kappaB and caspase-2 in response to genotoxic stress. *Oncogene*.

[16] Yeung B. H. Y., Law A. Y. S., Wong C. K. C. (2012). Evolution and roles of stanniocalcin. *Molecular and Cellular Endocrinology*.

[17] Chang A. C., Janosi J., Hulsbeek M. (1995). A novel human cDNA highly homologous to the fish hormone stanniocalcin. *Molecular and Cellular Endocrinology*.

[18] Ma X., Gu L., Li H. (2015). Hypoxia-induced overexpression of stanniocalcin-1 is associated with the metastasis of early stage clear cell renal cell carcinoma. *Journal of Translational Medicine*.

[19] Chan K. K., Leung C. O., Wong C. C. (2017). Secretory Stanniocalcin 1 promotes metastasis of hepatocellular carcinoma through activation of JNK signaling pathway. *Cancer Letters*.

[20] Shah N., Brown M. (2019). The sly oncogene: FOXA1 mutations in prostate cancer. *Cancer Cell*.

[21] Bernardo G. M., Keri R. A. (2012). FOXA1: a transcription factor with parallel functions in development and cancer. *Bioscience Reports*.

[22] Karpathiou G., Venet M., Mobarki M., Forest F., Chauleur C., Peoc'h M. (2017). FOXA1 is expressed in ovarian mucinous neoplasms. *Pathology*.

[23] Liu S., Zhang J., Zhu J., Jiao D., Liu Z. (2020). Prognostic values of EDNRB in triple-negative breast cancer. *Oncology Letters*.

[24] Fu W., Wu X., Yang Z., Mi H. (2019). The effect of miR-124-3p on cell proliferation and apoptosis in bladder cancer by targeting EDNRB. *Archives of Medical Science*.

[25] Zhang L., Luo B., Dang Y. W. (2019). The clinical significance of endothelin receptor type B in hepatocellular carcinoma and its potential molecular mechanism. *Experimental and Molecular Pathology*.

[26] Wang T., Liu H., Pei L. (2020). Screening of tumor-associated antigens based on Oncomine database and evaluation of diagnostic value of autoantibodies in lung cancer. *Clinical Immunology*.

[27] Lheureux S., Braunstein M., Oza A. M. (2019). Epithelial ovarian cancer: evolution of management in the era of precision medicine. *CA: a Cancer Journal for Clinicians*.

[28] Hennessy B. T., Coleman R. L., Markman M. (2009). Ovarian cancer. *Lancet*.

[29] Yang W. L., Gentry-Maharaj A., Simmons A. (2017). Elevation of TP53 autoantibody before CA125 in preclinical invasive epithelial ovarian cancer. *Clinical Cancer Research*.

[30] Qin J. J., Wang X. R., Wang P. (2014). Mini-array of multiple tumor-associated antigens (TAAs) in the immunodiagnosis of esophageal cancer. *Asian Pacific Journal of Cancer Prevention*.

[31] Janssens S., Tinel A., Lippens S., Tschopp J. (2005). PIDD mediates NF-kappaB activation in response to DNA damage. *Cell*.

[32] Bradley G., Tremblay S., Irish J. (2007). The expression of p53-induced protein with death domain (Pidd) and apoptosis in oral squamous cell carcinoma. *British Journal of Cancer*.

[33] Ji L., Zhang R., Chen J., Xue Q., Moghal N., Tsao M. S. (2019). PIDD interaction with KEAP1 as a new mutation-independent mechanism to promote NRF2 stabilization and chemoresistance in NSCLC. *Scientific Reports*.

[34] Liu G., Yang G., Chang B. (2010). Stanniocalcin 1 and ovarian tumorigenesis. *Journal of the National Cancer Institute*.

[35] Chang A. C., Doherty J., Huschtscha L. I. (2015). STC1 expression is associated with tumor growth and metastasis in breast cancer. *Clinical & Experimental Metastasis*.

[36] Fujiwara Y., Sugita Y., Nakamori S. (2000). Assessment of Stanniocalcin-1 mRNA as a molecular marker for micrometastases of various human cancers. *International Journal of Oncology*.

[37] Daniel A. R., Lange C. A. (2009). Protein kinases mediate ligand-independent derepression of sumoylated progesterone receptors in breast cancer cells. *Proceedings of the National Academy of Sciences of the United States of America*.

[38] Ismail R. S., Baldwin R. L., Fang J. (2000). Differential gene expression between normal and tumor-derived ovarian epithelial cells. *Cancer Research*.

[39] Park W. Y., Hong B. J., Lee J., Choi C., Kim M. Y. (2016). H3K27 demethylase JMJD3 employs the NF-*κ*B and BMP signaling pathways to modulate the tumor microenvironment and promote melanoma progression and metastasis. *Cancer Research*.

[40] Han J., Jeon M., Shin I., Kim S. (2016). Elevated STC-1 augments the invasiveness of triple-negative breast cancer cells through activation of the JNK/c-Jun signaling pathway. *Oncology Reports*.

[41] Tohmiya Y., Koide Y., Fujimaki S. (2004). Stanniocalcin-1 as a novel marker to detect minimal residual disease of human leukemia. *The Tohoku Journal of Experimental Medicine*.

[42] Ellard J. P., McCudden C. R., Tanega C. (2007). The respiratory effects of stanniocalcin-1 (STC-1) on intact mitochondria and cells: STC-1 uncouples oxidative phosphorylation and its actions are modulated by nucleotide triphosphates. *Molecular and Cellular Endocrinology*.

[43] Zhao F., Yang G., Feng M. (2020). Expression, function and clinical application of stanniocalcin-1 in cancer. *Journal of Cellular and Molecular Medicine*.

[44] Parolia A., Cieslik M., Chu S. C. (2019). Distinct structural classes of activating FOXA1 alterations in advanced prostate cancer. *Nature*.

[45] He S., Zhang J., Zhang W., Chen F., Luo R. (2017). FOXA1 inhibits hepatocellular carcinoma progression by suppressing PIK3R1 expression in male patients. *Journal of Experimental & Clinical Cancer Research*.

[46] Katoh M., Igarashi M., Fukuda H., Nakagama H., Katoh M. (2013). Cancer genetics and genomics of human FOX family genes. *Cancer Letters*.

[47] Gan H. Y., Li N., Zhang Q., Feng Z. Z. (2018). Silencing FOXA1 gene regulates liver cancer cell apoptosis and cell proliferation. *European Review for Medical and Pharmacological Sciences*.

[48] Urano M., Hirai H., Tada Y. (2018). The high expression of FOXA1 is correlated with a favourable prognosis in salivary duct carcinomas: a study of 142 cases. *Histopathology*.

[49] Reddy O. L., Cates J. M., Gellert L. L. (2015). Loss of FOXA1 drives sexually dimorphic changes in urothelial differentiation and is an independent predictor of poor prognosis in bladder cancer. *The American Journal of Pathology*.

[50] Wang L. L., Xiu Y. L., Chen X. (2017). The transcription factor FOXA1 induces epithelial ovarian cancer tumorigenesis and progression. *Tumour Biology*.

[51] Wang P., Qin J., Ye H., Li L., Wang X., Zhang J. (2019). Using a panel of multiple tumor-associated antigens to enhance the autoantibody detection in the immunodiagnosis of ovarian cancer. *Journal of Cellular Biochemistry*.

[52] Li L., Wang K., Dai L., Wang P., Peng X. X., Zhang J. Y. (2008). Detection of autoantibodies to multiple tumor-associated antigens in the immunodiagnosis of ovarian cancer. *Molecular Medicine Reports*.

